# Ventricular Assist Device implant (AB 5000) prototype cannula: *In vitro *assessment of MRI issues at 3-Tesla

**DOI:** 10.1186/1532-429X-10-23

**Published:** 2008-05-21

**Authors:** Frank G Shellock, Samuel Valencerina

**Affiliations:** 1 Keck School of Medicine, University of Southern California and Institute for Magnetic Resonance Safety, Education, and Research, Los Angeles, CA, USA; 2 University of Southern California, University Hospital, Los Angeles, CA, USA

## Abstract

**Purpose:**

To evaluate MRI issues at 3-Tesla for a ventricular assist device (VAD).

**Methods:**

The AB5000 Ventricle with a prototype Nitinol wire-reinforced In-Flow Cannula and Out-Flow Cannula attached (Abiomed, Inc., Danvers, MA) was evaluated for magnetic field interactions, heating, and artifacts at 3-Tesla. MRI-related heating was assessed with the device in a gelled-saline-filled, head/torso phantom using a transmit/received RF body coil while performing MRI at a whole body averaged SAR of 3-W/kg for 15-min. Artifacts were assessed for the main metallic component of this VAD (atrial cannula) using T1-weighted, spin echo and gradient echo pulse sequences.

**Results:**

The AB5000 Ventricle with the prototype In-Flow Cannula and Out-Flow Cannula attached showed relatively minor magnetic field interactions that will not cause movement *in situ*. Heating was not excessive (highest temperature change, +0.8°C). Artifacts may create issues for diagnostic imaging if the area of interest is in the same area or close to the implanted metallic component of this VAD (i.e., the venous cannula).

**Conclusion:**

The results of this investigation demonstrated that it would be acceptable for a patient with this VAD (AB5000 Ventricle with a prototype Nitinol wire-reinforced In-Flow Cannula and Out-Flow Cannula attached) to undergo MRI at 3-Tesla or less. Notably, it is likely that the operation console for this device requires positioning a suitable distance (beyond the 100 Gauss line or in the MR control room) from the 3-Tesla MR system to ensure proper function of the VAD.

## Introduction

A ventricular assist device (VAD) is used to provide temporary mechanical support for treatment of acute heart failure with the primary goal of producing rapid restoration of the circulation and stabilization of hemodynamics [1,2]. In 2003, the United States Food and Drug Administration approved the AB5000 Circulatory Support System (Abiomed, Inc., Danvers, MA) for temporary support of one or both sides of the natural heart in circumstances where the heart has failed, giving the patient's cardiovascular system the opportunity to rest and potentially recover – and providing surgeons the therapeutic flexibility necessary to determine the best endpoint for treatment [2]. The AB5000 Circulatory Support System is a paracorporeal device that can achieve pulsatile hemodynamic support up to 6 liters of flow per minute [2-4]. This device is increasingly used to manage patients because it offers the advantage of allowing patients to ambulate, which greatly assists in the recovery process [2-4].

The utilization of 3-Tesla MR systems for clinical applications, especially relative to cardiovascular examinations, is increasing worldwide [5,6]. Biomedical implants may pose risks and other issues to patients referred for 3-Tesla MRI procedures that include movement or dislodgement of the item and/or excessive heating of the device [5,6]. The current commercially available AB5000 Circulatory Support System includes a ferromagnetic, stainless steel, wire-reinforced in-flow cannula, which is likely to be problematic for a patient referred for an MRI procedure. For the purpose of creating a more acceptable AB5000 Circulatory Support System with respect to MRI, the approved cannula was exchanged with a prototype, Nitinol wire-reinforced in-flow cannula. In consideration of the growing use of the AB5000 Circulatory Support System as well as that of 3-Tesla MRI examinations, especially for cardiac MR procedure, the purpose of this investigation was to assess magnetic field interactions, heating, and artifacts to determine if it would be acceptable to scan a patient with this VAD using the modified in-flow cannula. Notably, it is likely that the operation console for this device requires positioning a suitable distance (beyond the 100 Gauss line or in the MR control room) from the 3-Tesla MR system to ensure proper function of the VAD.

## Materials and methods

### Ventricular Assist Device

**Figure 1:**
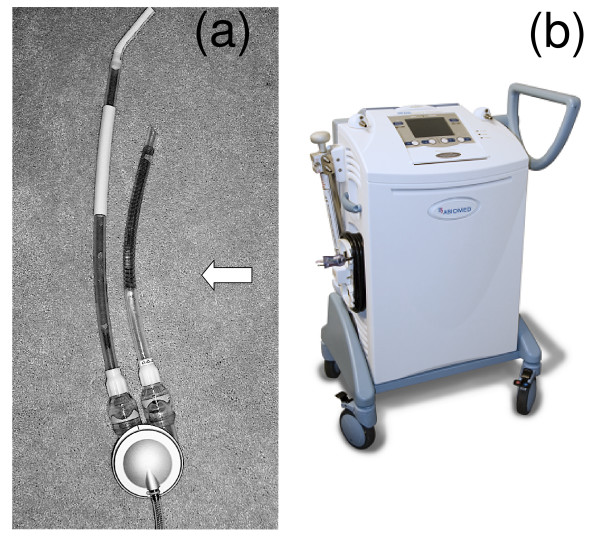
**a**. **The AB5000 Ventricle with the prototype Nitinol wire-reinforced In-Flow Cannula and Out-Flow Cannula attached that underwent testing for magnetic field interactions, heating, and artifacts at 3-Tesla.** Note the prototype Nitinol wire-reinforced In-Flow Cannula atrial cannula (arrow). This is the only metallic portion of this ventricular assist device that is implanted. **b**. The AB5000 Circulatory Support System (Abiomed, Inc., Danvers, MA). Note the console component of this ventricular assist device.

The AB5000 Circulatory Support System (Abiomed, Inc., Danvers, MA) is a ventricular assist device (VAD) comprised of the AB5000 Ventricle, the stainless steel wire-reinforced In-Flow Cannula and the Out-Flow Cannula, and the AB5000 Console (Figure 1). The AB5000 Ventricle is a pneumatically driven blood pump located next to the body (paracorporeal) and attaches to the heart via the In-Flow Cannula and the Out-Flow Cannula which, as tubes, pass through the patient's skin and connect directly to the AB5000 Console (Figure 1). The AB5000 Ventricle is vacuum assisted technology with clear housing to allow clinicians a view into the device. Materials for the AB5000 Ventricle include anodized aluminum, titanium, epoxy, and Class VI medical grade polycarbonate. The cannulas are made from clear, medical grade plastic, with stainless steel metal added to the atrial cannula as a means of reinforcement (note that the metal reinforced part of the atrial cannula is insulated and, thus, would not be in direct contact with patient tissue, *in situ*).

Notably, for this investigation, the commercially available ferromagnetic, stainless steel wire-reinforced cannula was replaced with a prototype Nitinol wire-reinforced cannula. MRI testing at 3-Tesla was performed on the AB5000 Ventricle with the prototype In-Flow Cannula and Out-Flow Cannula attached.

The other component of the AB5000 Circulatory Support System is the AB5000 Console, which is a software-driven device that operates one or two AB5000 Ventricles. The AB5000 Ventricle attaches to this console via a 6-foot drive-line (i.e., plastic tube) that has a metallic connector. This console automatically adjusts its settings to the outputs of the natural heart, allowing the heart to contribute to the pumping function but providing it the necessary assistance to rest and recover. The console incorporates several systems to ensure safe operation, including back up power from internal batteries, alarms that inform the clinicians as to status of operation and back-up internal software systems to ensure safe operation. The console comes with a portable cart that can serve as a means in the event that patient transport is required or as a walker, allowing the patient to ambulate about the hospital. In case of an emergency, a hand-operated pump allows continuous support.

### Magnetic Field Interactions

The AB5000 Ventricle with prototype Nitinol wire-reinforced In-Flow Cannula and Out-Flow Cannula attached was evaluated for translational attraction and torque in association with a shielded, 3-Tesla MR system (Excite, Software G3.0-052B, General Electric Healthcare, Milwaukee, WI; active-shielded, horizontal field scanner).

#### Translational Attraction

To evaluate translational attraction for the Ventricle with prototype Nitinol wire-reinforced In-Flow Cannula and Out-Flow Cannula attached, the deflection angle was measured using a previously described technique [7-15]. The device was connected to a test fixture to determine the deflection angle in the 3-Tesla MR system. The test fixture consisted of a sturdy structure capable of holding the device in a proper position. The test fixture incorporated a protractor with 1-degree graduated markings [7-15]. The Ventricle with prototype Nitinol wire-reinforced In-Flow Cannula and Out-Flow Cannula attached was suspended on the apparatus by a lightweight string (20-cm in length; weight, less than 1% of the weight of the device) that was fixed at the 0-degree indicator of the protractor. Deflection angles were assessed at the point of the highest spatial magnetic gradient for the 3-Tesla MR system [7-15]. The highest spatial gradient for this scanner is 720 gauss/cm and it occurs at a position that is 74-cm from isocenter [9-12]. The deflection angle from the vertical direction to the nearest 1-degree was measured three times for the device and an average value was calculated [7-15].

#### Torque

Torque related to exposure to the 3-Tesla MR system was assessed for the Ventricle with prototype Nitinol wire-reinforced In-Flow Cannula and Out-Flow Cannula attached utilizing a previously described, qualitative technique [9,10]. This involved the use of a flat plastic device with a millimeter grid [9,10]. The device was placed on the test apparatus in an orientation that was 45-degrees relative to the static magnetic field of the 3-Tesla MR system [9,10]. The test apparatus with device was then positioned in the center of the scanner, where the effect of torque is the greatest and observed for possible alignment or rotation relative to the 3-Tesla static magnetic field [9,10]. The Ventricle with prototype Nitinol wire-reinforced In-Flow Cannula and Out-Flow Cannula attached was then moved 45 degrees relative to its previous position and again observed for alignment or rotation [9,10]. This process was repeated to encompass a full 360-degrees rotation of positions for the device. The following qualitative scale was applied to the results [9,10]: 0, no torque; +1, mild or low torque, the device slightly changed orientation but did not align to the magnetic field; +2, moderate torque, the device aligned gradually to the magnetic field; +3, strong torque, the device showed rapid and forceful alignment to the magnetic field; +4, very strong torque, the device showed very rapid and very forceful alignment to the magnetic field [9,10].

### MRI-Related Heating

#### Phantom and Experimental Set-up

MRI-related heating at 3-Tesla/128-MHz was assessed for the Ventricle with prototype Nitinol wire-reinforced In-Flow Cannula and Out-Flow Cannula attached. This procedure used a plastic phantom that approximated the dimensions of the human head and torso, as follows [13,16,17]: head portion – width, 16.5-cm; length, 29.2-cm; height, 16.5-cm; torso portion – width, 43.2-cm; length, 61.0-cm; height, 16.5-cm. The phantom was filled to a depth of 10-cm with a gelling agent (hydroxyetheyl-cellulose) in an aqueous solution (91.48% H_2_O) along with 0.12% NaCl [8-13,16,17].

**Figure 2:**
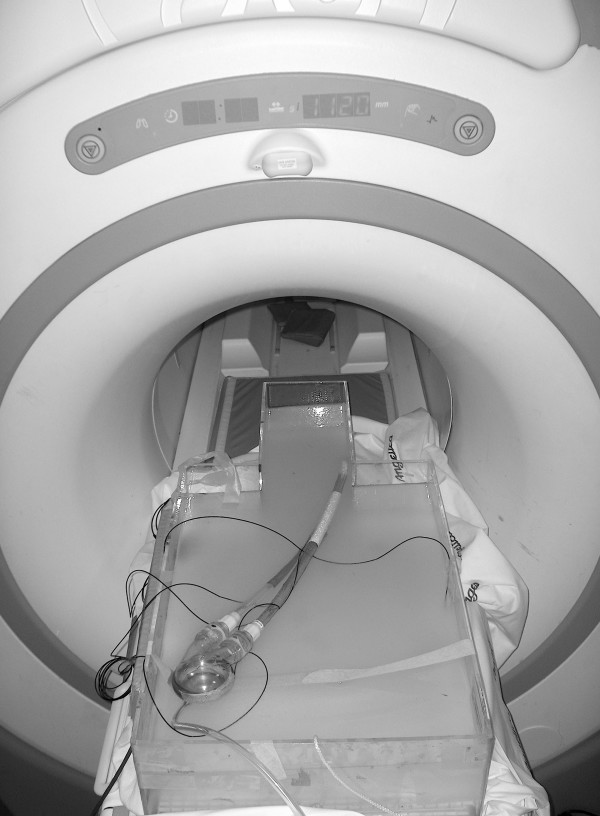
**Experimental set up showing the 3-Tesla MR system and head/torso phantom used for the evaluation of MRI-related heating for the Ventricle with the prototype Nitinol wire-reinforced In-Flow Cannula and Out-Flow Cannula.** Note the cables going to the fluoroptic thermometry probes of the thermometry system.

The position of the Ventricle with prototype Nitinol wire-reinforced In-Flow Cannula and Out-Flow Cannula attached was maintained to simulate its intended *in vivo *use in the head/torso phantom using highly porous, 3-M Micropore™ Tape (3-M Company, St. Paul, MN) that does not affect temperature recordings nor add insulative material to the device (i.e., unpublished observations obtained from pilot experiments) (Figure 2). Importantly, the metal portions of the Ventricle with prototype Nitinol wire-reinforced In-Flow Cannula and Out-Flow Cannula attached were positioned so that they were immersed in the gelled saline of the head/torso phantom (Figure 2). Because this experimental set-up lacks "blood flow", it simulates an extreme condition used to assess MRI-related heating for the Ventricle with prototype Nitinol wire-reinforced In-Flow Cannula and Out-Flow Cannula attached.

#### Temperature Recording System and Placement of Thermometry Probes

Temperature measurements were obtained using a fluoroptic thermometry system (Model 3100, LumaSense Technologies, Santa Clara, CA). The fluoroptic thermometry probes (0.5-mm in diameter) were positioned on the Ventricle with prototype Nitinol wire-reinforced In-Flow Cannula and Out-Flow Cannula attached to record sites that would generate the greatest heating during MR imaging (i.e., based on pilot experiments that were conducted), as follows: probe #1, sensor portion of the probe placed in contact with metal, posterior portion of the VAD; probe #2, sensor portion of the probe placed in contact with metal, side portion of the VAD; probe #3, sensor portion of the probe placed in contact with prototype Nitinol wire-reinforced atrial cannula (Figure 2). All other portions of the Ventricle with prototype Nitinol wire-reinforced In-Flow Cannula and Out-Flow Cannula attached involved nonmetallic, non-conducting materials and, as such, would not be subjected to MRI-related heating. The connector component of the Ventricle was positioned and taped to the end of the MR system table (i.e., approximately 6-feet from the transmit/receive RF body coil). Thus, this connector will not heat in relation to MRI. A thermometry probe was placed in the phantom at a position removed (close to the opposite side of the head/torso phantom) from the Ventricle with prototype Nitinol wire-reinforced In-Flow Cannula and Out-Flow Cannula attached but within the area of MR imaging, to record a reference temperature during the heating experiment (probe #4). The positions of the thermometry probes were inspected and verified immediately before and after the MRI-related heating experiment.

#### MRI Conditions

MR imaging was performed at 3-Tesla/128-MHz using a transmit RF body coil. MRI parameters were selected using a fast spin echo pulse sequence to generate a relatively high level of radiofrequency (RF) energy [9-12], producing an MR system reported, whole body averaged specific absorption rate (SAR) of 3.0-W/kg for 15-min. The land-marking position (i.e., the center position or anatomic region for the MR imaging procedure) and section locations were selected to encompass the entire area of the Ventricle with prototype Nitinol wire-reinforced In-Flow Cannula and Out-Flow Cannula attached.

#### Experimental Protocol

The Ventricle with prototype Nitinol wire-reinforced In-Flow Cannula and Out-Flow Cannula attached was positioned in the head/torso phantom as previously described. The fluoroptic thermometry system was calibrated and the fluoroptic thermometry probes were applied. The head/torso phantom was filled with the gelled-saline and allowed to equilibrate to the environmental temperature for approximately 24-hours. The room temperature and the temperature of the bore of the MR system were at a constant level throughout the MRI-related heating experiment. After recording baseline temperatures (5-min.), MR imaging was performed for 15-min. with temperatures recorded at 20-sec. intervals. This highest temperature changes recorded by the fluoroptic thermometry probes are reported herein.

### Artifacts

MR imaging artifacts were assessed at 3-Tesla for the Ventricle with prototype Nitinol wire-reinforced In-Flow Cannula and Out-Flow Cannula attached. This test was accomplished by performing MR imaging with the device placed in a gadolinium-doped, saline filled plastic phantom [8-13]. Notably, most of the metal parts for this VAD remain outside of the patient (i.e., it is paracorporeal) with only the distal aspects of the prototype Nitinol wire-reinforced In-Flow Cannula and Out-Flow Cannula implanted. Thus, because the primary portion of this VAD that would cause artifact during implantation, due to its material content is the metal reinforced atrial cannula, the artifact evaluation emphasized this component part. The prototype Nitinol wire-reinforced atrial cannula was attached to a plastic frame to facilitate positioning and MR imaging within this phantom.

MR imaging was conducted using a 3-Tesla MR system (Excite, Software G3.0-052B, General Electric Healthcare, Milwaukee, WI), a transmit/receive RF body coil, and the following pulse sequences: (1) T1-weighted, spin echo pulse sequence; repetition time, 500-msec; echo time, 20-msec; matrix size, 256 × 256; section thickness, 10-mm; field of view, 42-cm; number of excitations, 2; bandwidth; 16 kHz (2) Gradient echo (GRE) pulse sequence; repetition time, 100-msec; echo time, 15-msec; flip angle, 30 degrees; matrix size, 256 × 256; section thickness, 10-mm; field of view, 42-cm; number of excitations, 2; bandwidth, 16 kHz, similar to previously described assessments of artifacts [8-13]. The imaging planes were oriented to encompass the long axis and short axis of the prototype Nitinol wire-reinforced atrial cannula. The frequency encoding direction was parallel to the plane of imaging. The image locations obtained through the prototype Nitinol wire-reinforced atrial cannula were selected from multiple "scout" MR images to represent the largest or worst-case artifacts for this device. Planimetry software was used to measure (accuracy and resolution ± 10%) the cross-sectional area of the largest artifact size for the prototype Nitinol wire-reinforced atrial cannula of the Ventricle for each pulse sequence, and for each orientation of the section location. The image display parameters (i.e., window and level settings, magnification, etc.) were carefully selected and used in a consistent manner to facilitate valid measurements of artifact size [8-13].

## Results

**Figure 3:**
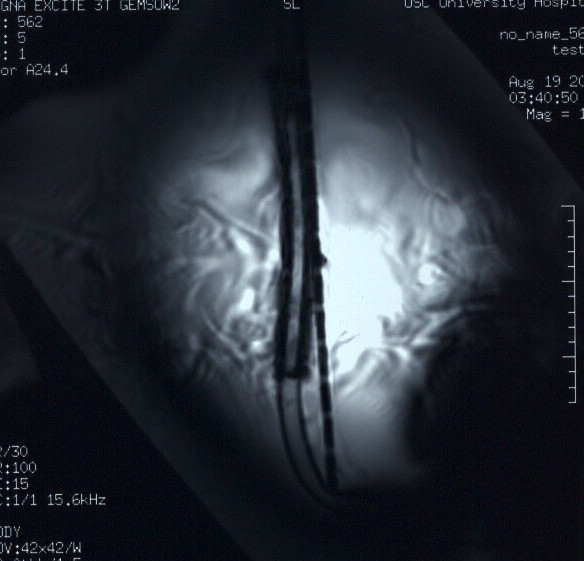
MRI artifact associated with the prototype Nitinol wire-reinforced atrial cannula used with the AB5000 Ventricle (gradient echo pulse sequence; TR/TE, 100-msec/15-msec; flip angle, 30 degrees; matrix size, 256 × 256; section thickness, 10-mm; field of view, 42-cm; long axis imaging plane).

The average deflection angle for the AB5000 Ventricle with prototype Nitinol wire-reinforced In-Flow Cannula and Out-Flow Cannula was 22-degrees and the qualitatively measured torque was 0, no torque. Findings for the MRI-related heating assessment indicated that the highest temperature change recorded by probe #1 was +0.7°C, +0.8°C for probe #2, and +0.5°C for probe #3. The highest temperature change measured by the reference probe was +0.5°C. Artifact test results are summarized in Table 1. The artifacts associated with the prototype Nitinol wire-reinforced atrial cannula were seen as signal voids that were slightly larger than the size and shape of the metallic component for this device, with the GRE pulse sequence producing larger artifacts than the T1-weighted, spin echo pulse sequence. Figure 3 shows an example of artifact associated with the prototype Nitinol wire-reinforced atrial cannula as seen on a GRE pulse sequence.

**Table 1:** Artifact size for the atrial cannula of the AB5000 Ventricle.

**Pulse Sequence**	**Plane Orientation**	**Signal Void (mm^**2**^)**
T1-SE	long axis	2,373
	short axis	190
GRE	long axis	4,457
	short axis	554

(Imaging plane relative to the atrial cannula which is the implanted portion of the AB5000 Ventricle that is reinforced by metal; T1-SE, T1-weighted spin echo; GRE, gradient echo)

## Discussion

The AB5000 Circulatory Support System is intended to treat patients suffering from reversible ventricular dysfunction. Typical patients that would be appropriate for this VAD have undergone successful cardiac surgery and subsequently developed low cardiac output, or have suffered from acute cardiac disorders leading to hemodynamic instability [1-4]. In all probability, considering the increased utilization of the AB5000 as well as the importance of 3-Tesla MRI for diagnostic imaging [5,6], a patient with a VAD may be referred for an MRI examination in order to assess a pre-existing health problem or in the event a new condition develops. As such, it is necessary to determine the MRI-related issues (i.e., magnetic field interactions, MRI-related heating, and artifacts) for these types of devices.

The average deflection angle at 3-Tesla for the Ventricle with prototype Nitinol wire-reinforced In-Flow Cannula and Out-Flow Cannula was 22-degrees and the qualitatively measured torque was 0, no torque. The 22-degree deflection angle measurement should be considered in view of the deflection angle measurement recommendation provided by the ASTM International [17], which states: "If the implant deflects less than 45°, then the magnetically induced deflection force is less than the force on the implant due to gravity (its weight). For this condition, it is assumed that any risk imposed by the application of the magnetically induced force is no greater than any risk imposed by normal daily activity in the Earth's gravitational field." Thus, 22-degrees is substantially less than 45° and, therefore, poses no hazard to the patient in a 3-Tesla MRI environment. Thus, relatively minor magnetic field interactions exist overall for this implant. Notably, the primary cause of magnetic field interactions for this VAD was the connector component, which is not implanted in the patient, but rather connected to the control console, which should be positioned a suitable distance from the MR system to permit proper operation.

Proper placement of the console during an MRI procedure may be accomplished by attaching extension tubing to the end of the 6-foot connector, which would permit the console to be positioned an appropriate distance from the MR system (i.e., outside of the entrance) or in the MR control room. (Note: Tests conducted by Abiomed, Inc. indicated that the addition extension tubing, on average, would decrease the pneumatic function by 0.3 l/m, which is not a substantial level). While another alternative to the use of the console is to utilize the hand-operated pump, this is not recommended for the AB5000 Ventricle during an MRI procedure. The possible presence of RF interference generated by the console and the impact on MRI is unknown.

MRI-related heating for the AB5000 Ventricle with prototype Nitinol wire-reinforced In-Flow Cannula and Out-Flow Cannula was equal to or less than +0.8°C. Excessive MRI-related heating may occur in implants and devices made from metallic materials [6-13], but this only tends to occur for those items that have a certain length or that have closed loops of a relatively large diameter [6-13]. Importantly, this VAD has relatively short length metal parts and substantial non-conducting, nonmetallic materials in its construction. These nonmetallic, non-conducting materials effectively act to isolate and to insulate the metallic materials of this implant and, thus, do not pose an additional MRI-related heating risk for a patient undergoing a 3-Tesla MRI procedure.

With respect to the findings for artifacts, the main component of the AB5000 that causes artifacts on MR images is the prototype Nitinol wire-reinforced atrial cannula. Using T1-weighted, spin echo and gradient echo pulse sequences, the extent of the artifacts observed at 3-Tesla was considered to be relatively small in relation to the size and shape of this prototype cannula. However, the associated artifact may still affect the diagnostic use of MR imaging if the area of interest is close to this metallic region. Optimization of pulse sequence parameters to minimize artifact size is, therefore, recommended.

## Summary and Recommendations

The results of this investigation demonstrated that it would be acceptable for a patient with this VAD (AB5000 Ventricle with prototype Nitinol wire-reinforced In-Flow Cannula and Out-Flow Cannula attached) to undergo MRI at 3-Tesla or less. Notably, the Nitinol cannula is currently not commercially available and it is likely that the operation console for this device requires positioning a suitable distance (beyond the 100 Gauss line or in the MR control room) from the 3-Tesla MR system to ensure proper function of the VAD. As such, this should be taken into consideration when preparing to scan a patient with the AB5000 Circulatory Support System.

## Competing interests

The authors declare that they have no competing interests.

## Authors' contributions

FGS made substantial contributions to the conception and design of this study, the acquisition of the data, the analysis and interpretation of data, the drafting of the manuscript and revising it critically for important intellectual content, and gave final approval of the version to be published. SV made substantial contributions to the conception and design of this study, participated in the collection of the data, and gave final approval of the version to be published.
